# Standard Lymphadenectomy for Esophageal and Lung Cancer: Variability in the Number of Examined Lymph Nodes Among Pathologists and Its Survival Implication

**DOI:** 10.1245/s10434-022-12826-0

**Published:** 2022-11-25

**Authors:** Mikko Uimonen, Olli Helminen, Jan Böhm, Johanna Mrena, Eero Sihvo

**Affiliations:** 1 Department of Surgery, Central Finland Hospital Nova, Jyväskylä, Finland; 2 Surgery Research Unit, Medical Research Center Oulu, Oulu University Hospital and University of Oulu, Oulu, Finland; 3 Department of Pathology, Central Finland Hospital Nova, Jyväskylä, Finland; 4 Faculty of Medicine and Health Techologies, Tampere University, Tampere, Finland

## Abstract

**Aim:**

We compared variability in number of examined lymph nodes between pathologists and analyzed survival implications in lung and esophageal cancer after standardized lymphadenectomy.

**Methods:**

Outcomes of 294 N2 dissected lung cancer patients and 132 2-field dissected esophageal cancer patients were retrospectively examined. The primary outcome was difference in reported lymph node count among pathologists. Secondary outcomes were overall and disease-specific survival related to this count and survival related to the 50% probability cut-off value of detecting metastasis based on the number of examined lymph nodes.

**Results:**

The median number of examined lymph nodes in lung cancer was 13 (IQR 9–17) and in esophageal cancer it was 22 (18–29). The pathologist with the highest median number of examined nodes had > 50% higher lymph node yield compared with the pathologist with the lowest median number of nodes in lung (15 vs. 9.5, *p* = 0.003), and esophageal cancer (28 vs. 17, *p* = 0.003). Survival in patients stratified by median reported lymph node count in both lung (adjusted RMST ratio < 14 vs. ≥ 14 lymph nodes 0.99, 95% CI 0.88–1.10; *p* = 0.810) and esophageal cancer (adjusted RMST ratio < 25 vs. ≥ 25 lymph nodes 0.95, 95% CI 0.79–1.15, *p* = 0.612) was similar. The cut-off value for 50% probability of detecting metastasis by number of examined lymph nodes in lung cancer was 15.7 and in esophageal cancer 21.8. When stratified by this cut-off, no survival differences were seen.

**Conclusion:**

The quality of lymphadenectomy based on lymph node yield is susceptible to error due to detected variability between pathologists in the number of examined lymph nodes. This variability in yield did not have any survival effect after standardized lymphadenectomy.

**Supplementary Information:**

The online version contains supplementary material available at 10.1245/s10434-022-12826-0.

The presence of lymph node metastasis is an indicator of advanced disease in solid cancers. In esophageal cancer, the stage and prognosis are related to the number of lymph node metastases.^1^ This number-based classification has been suggested to improve the current location-based N1 vs. N2 classification in lung cancer as well.^2^

Nodal involvement is the major determinant of the need for oncological treatment in both lung and esophageal cancer. In lung cancer, patients with nodal disease in surgical specimens require adjuvant chemotherapy.^1^ Neoadjuvant chemo- or chemoradiotherapy is the most common mode of multimodality therapy in esophageal cancer.^1^ In this disease, when there is incidental nodal involvement in cT1-2N0-operated patients, adjuvant therapy has been shown to be associated with improved survival.^3^

As the probability of detecting metastasis increases by the number of examined lymph nodes, the thoroughness of lymphadenectomy has been considered to be a key aspect of the quality of surgical treatment in lung and esophageal cancer. In both cancers, the number of examined lymph nodes, i.e., lymph node yield, has been shown to be associated with survival.^4,5^ Even after comprehensive lymphadenectomy yielding the maximal number of nodes, possible metastatic disease is ultimately diagnosed or, in more unfortunate cases, left unnoticed by the pathologist examining the resected tissue sample. A high interobserver variability between pathologists in the number of examined lymph nodes in colorectal and bladder cancer has been reported.^6–10^ The difference in this variability between pathologists after mediastinal lymphadenectomy in lung cancer, or 2-field lymphadenectomy in esophageal cancer and its association with long-term survival is poorly known.

This study aimed to compare the number of examined lymph nodes between pathologists and further analyze its association with survival in lung and esophageal cancer patients who have undergone standardized lymphadenectomy.

## Methods

### Patients and Data Collection

All patients treated due to primary lung or esophageal cancer in Central Finland Central Hospital between January 1, 2013 and December 31, 2021 were eligible for this cohort study. Study patients were identified from the prospective surgical database. All surgically treated esophageal cancer patients, except three salvage cases, were included in the study (*n* = 132). Included patients underwent a standard 2-field lymphadenectomy with extension of lymphadenectomy to the right upper mediastinum only in highly selected cases. In order to homogenize the effect of surgery, only lung cancer patients with systematic N2 dissection (294, 88.3%) were included. The reason for exclusion was systematic N2 nodal sampling instead of formal lymphadenectomy in 23 patients. The reasons for less-limited sampling and exclusion of 15 patients included the following: high surgical risk due to poor exercise capacity, significant comorbidity load or significant comorbidities in 8 patients; a second primary tumor on the same side after previous lymphadenectomy in 3 patients; pure ground-glass opacity in 2 patients; unstable hemodynamics in 1 patient; and operation considered to be due to lung metastases in 1 patient. There were no missing data.

For both lung and esophageal cancer, the 8th edition of the TNM classification was used for staging, including recoding of older versions if necessary. Preoperative patient evaluation included guideline-based use of PET-CT and invasive staging.^1^ Physical evaluation was systematic physical performance testing, described in detail earlier.^11,12^ For survival data, medical records and the Death Registry from Statistics Finland was used. The follow-up ended December 31, 2021. The study was approved by the local hospital districts.

### Lymphadenectomy

All esophageal cancer patients underwent either totally minimally invasive (n = 90.2%) or hybrid (9.8%) Ivor Lewis (*n* = 88.6%) or McKeown (11.4%) esophagectomy with en bloc lymphadenectomy. The technique of 2-field lymphadenectomy has been described previously in detail.^13^ N2 lymph node dissection in lung cancer surgery was performed with the same principles as during either open or thoracoscopic surgery. The rate of video-assisted thoracoscopic surgery (VATS) was 84.9%. The proportion of right-sided operations was 56.7%. During right-sided operations, in addition to the hilar nodes, mediastinal stations 2R and 4R were removed via the en bloc surgery. Also, separate removal of stations 7, 8, and 9 was a routine part of the surgery. On the left side, the dissections of stations 7, 8, and 9 were performed similarly. The dissection in the left upper mediastinum included en bloc removal of stations 5 and 6 nodes, as well as separate removal of distal 4L nodes.

### Tissue Sampling

During the study, a single surgeon performed all lung and esophageal cancer operations with the standardized lymphadenectomy technique described above. Samples after lymphadenectomy were handled in a constant manner throughout the study period. On the back table, a senior surgeon separated the removed en bloc esophageal specimen nodal stations using a modified nomenclature from the Japanese Classification.^13^ These detached stations were submerged in separate formalin jars. The removed N2 stations in lung cancer were numbered according to international guidelines and placed in formalin. N1 nodes around the hilar structures of the lung specimens were dissected by the operating surgeon and submerged in formalin, as well. Further dissection was performed by the pathologist. A total of seven senior pathologists evaluated the samples (both lung and esophageal cancer samples).

Lung or esophageal tissue arrived at the pathology laboratory as fresh tissue, whereas removed lymph nodes were placed in dishes containing 10% formalin. After at least a 24-h fixation period, lung or esophageal tissues were examined by macroscopic dissection. Tumor dimensions and resection margins were recorded and tissue samples were taken from representative areas for histological evaluation. In order to assess lymph node status, all lymph nodes either from the vicinity of a primary tumor or from removed nodal stations were dissected. For histological examination, one representative piece of tissue was taken from each lymph node to assess the lymph node yield and possible metastases. Both the number of examined and metastatic lymph nodes were stated in the separate histology report. In this report, tumors were classified according to the most recent pTNM classification.

Central Finland Central Hospital pathology department had quality assurance acquired from Labquality Oy until the end of 2018. From the beginning of 2019, laboratory processes have been conducted according to ISO 15189:2013 requirements, and the laboratory will be acquiring quality accreditation from a national provider (FINAS) within 2023.

### Outcomes

The primary outcome was the difference in the examined number of lymph nodes among pathologists. The secondary outcomes were overall and disease-specific survival related to this count and survival related to the 50% probability cut-off value of detecting metastasis by the number of examined lymph nodes.

### Statistical Analysis

The patient characteristics are presented as means and standard deviations (SD), as medians and inter-quartile ranges (IQR), or counts and percentages. The number of lymph nodes examined by pathologists was assessed and compared between individual pathologists by using the Kruskal Wallis test.

Survival analysis was conducted separately among lung and esophageal cancer patients. Kaplan-Meier estimate curves were drawn and compared by log-rank test. To ensure the stability of Kaplan-Meier estimates, a follow-up time of 6 years was determined so that at the end of the follow-up, the number of patients at risk was more than 10% of the patients in the original sample. Restricted mean survival time (RMST) analysis was conducted to compare mean survival time within the follow-up period.^14^ Due to differing patient characteristics between lung and esophageal cancer patients, covariates were selected separately for lung and esophageal cancer patients. In lung cancer, the RMST ratio was adjusted by sex, age, body mass index, clinical stage of cancer (stage 1 vs. 2 vs. 3–4), physical condition (ability to walk up the stairs less than 10 m vs. 10–14 m vs. more than 14 m), Charlson comorbidity index (0–1 vs. 2–4 vs. 5 or over), and open versus video-assisted surgery. In esophageal cancer, RMST ratio was adjusted by sex, age, body mass index, clinical stage of cancer (stage 1 vs. 2 vs. 3–4), physical condition (ability to walk up the stairs less than 14 m vs. more than 14 m) and Charlson comorbidity index (0–1 vs. 2 or over).

For the survival analysis, patients were first divided into subgroups by thoroughness of pathologists. Thoroughness was determined according to the median number of examined lymph nodes. In lung cancer, pathologists examining a median of 14 or more lymph nodes were considered to be thorough, and in esophageal cancer a median of 25 or more were considered to be thorough. Survival between patients below and above these cut-offs was compared.

For the second survival analysis, the cut-off value for 50% probability of detecting metastasis was determined by predictive logistic modeling. Logistic regression analysis was performed by setting detection of metastasis (yes vs. no) as a dependent variable and the number of examined lymph nodes as an independent variable. The estimated cut-off value was adjusted by the rate of observed metastases and was determined separately for lung and esophageal cancer. After determining the cut-off, patients were divided into two groups by the cut-off and survival was compared between these subgroups.

Statistical analysis was performed using R (version 4.1.2) statistical software.

## Results

**Fig. 1:**
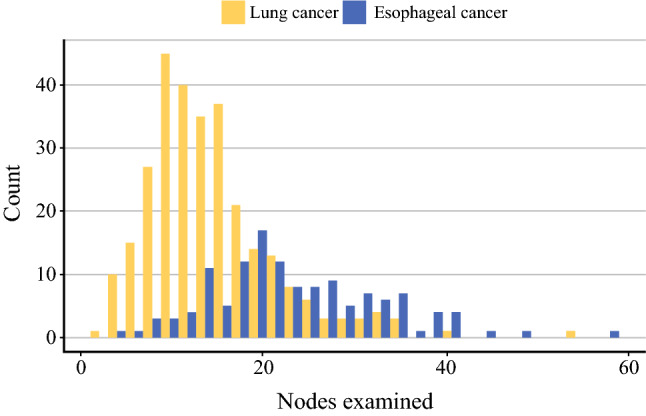
Distribution by the number of examined lymph nodes

The final number of patients included 294 lung and 132 esophageal cancer patients, totaling 426 (Table 1). Among these patients, the median number of examined lymph nodes was higher in esophageal cancer patients [22 (IQR 18–29) vs. 13 (9–17); *p* < 0.001; Fig. 1]. Median follow-up was 2.7 (IQR 1.3–4.8) years. Overall survival, and more evidently disease-specific survival in lung cancer, was dependent on the pN-stage with improved survival in lower-stage patients (Supplementary Figs. 1A and B, 2A and B).

**Table 1:** Patient characteristics

	Lung cancer	Esophageal cancer
N	294	132
Age, mean (SD)	69.3 (8.8)	66.4 (11.0)
Sex male, n (%)	185 (62.9)	98 (74.2)
BMI, mean (SD)	26.3 (4.57)	25.8 (4.93)
Charlson comorbidity index, *n* (%)		
0–1	158 (53.7)	102 (77.3)
2–4	124 (42.2)	29 (22.0)
5–8	15 (5.1)	1 (0.8)
Clinical stage, *n* (%)		
I	203 (69.0)	28 (21.2)
II	60 (20.4)	49 (37.1)
III	30 (10.2)	54 (40.9)
IV	1 (0.3)	1 (0.8)
Physical performance (stair climbing) *n* (%)		
Less than 10 m	18 (6.1)	–
10–14 m	33 (11.2)	11 (8.3)
14 m or more	243 (82.7)	121 (91.7)
Surgical approach, *n* (%)		
Open	42	–
VATS	252	–
Lymph node count, mean (SD)	14.2 (7.2)	23.4 (9.3)

### Lung Cancer

**Fig. 2:**
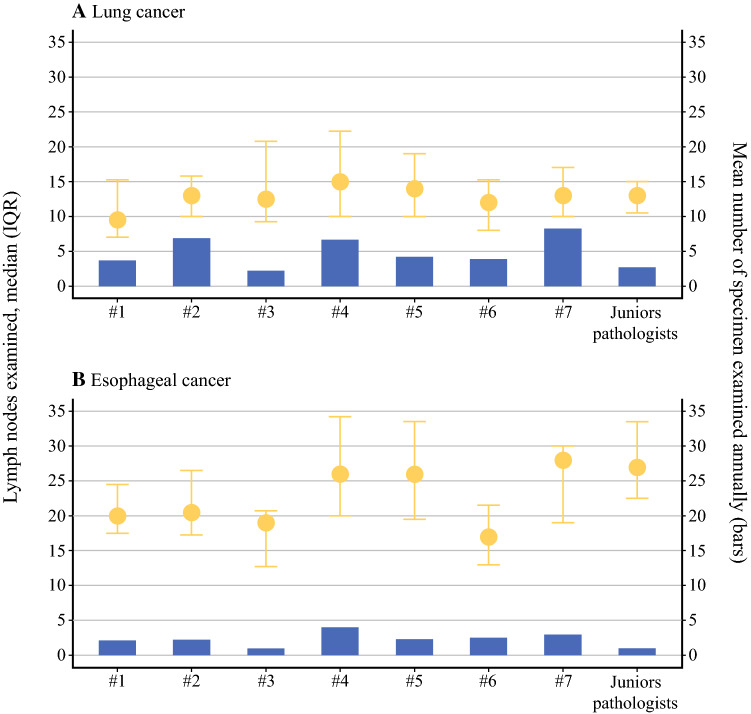
Median number of examined lymph nodes by pathologists. *Whiskers* show inter-quartile range and *bars* show mean number of examined specimens annually

Of seven pathologists, two had a median of more than 14 examined lymph nodes (Fig. 2). In the number of examined lymph nodes, differences were statistically significant between pathologists with the lowest and the highest median lymph node count (*p* = 0.026). The pathologist with the highest median number of examined nodes had 57.8% higher lymph node yield compared with the pathologist with the lowest median number of nodes (15 vs. 9.5, *p* = 0.003). No differences were detected in patient demographics or surgical treatment between patients whose specimens were examined by pathologists having a high lymph node count compared with patients whose specimens were examined by pathologists having a low lymph node count (Supplementary Table 1).

**Fig. 3:**
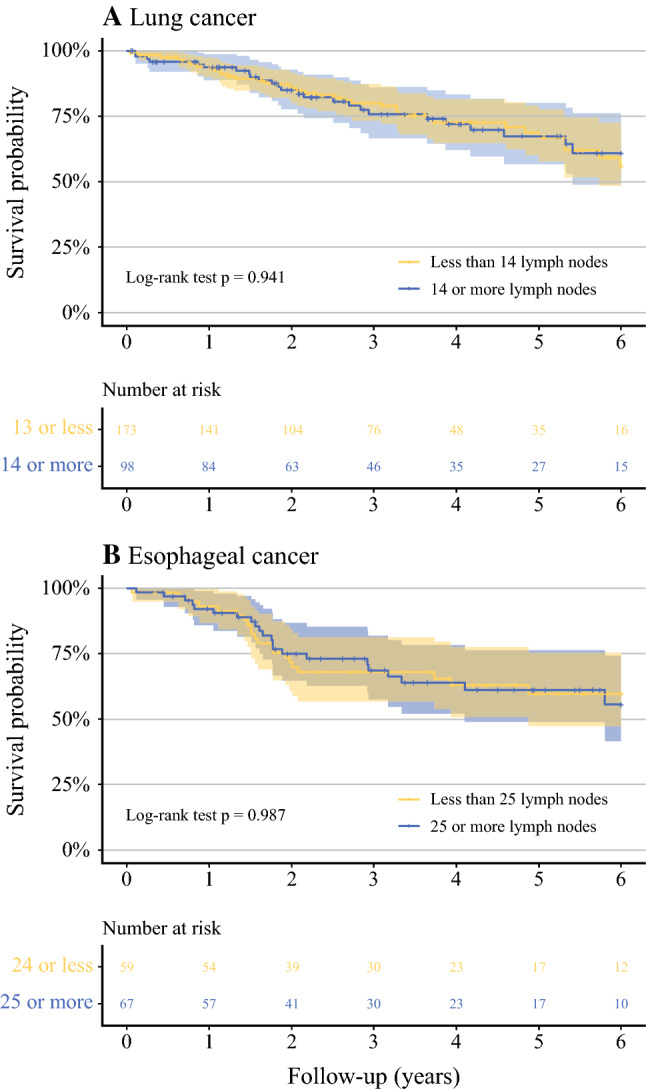
**A** and **B** Kaplan-Meier survival curves of lung and esophageal cancer patients stratified by median of examined lymph nodes by pathologists

Survival analysis in subgroups by the thoroughness of the pathologists (< 14 lymph nodes vs. ≥ 14 lymph nodes) showed equal survival (log-rank *p* = 0.941, Fig. 3A). Further, adjusted RMST ratio for < 14 vs. ≥ 14 lymph nodes was 0.99 (95% CI 0.88–1.10, *p* = 0.810).

**Fig. 4:**
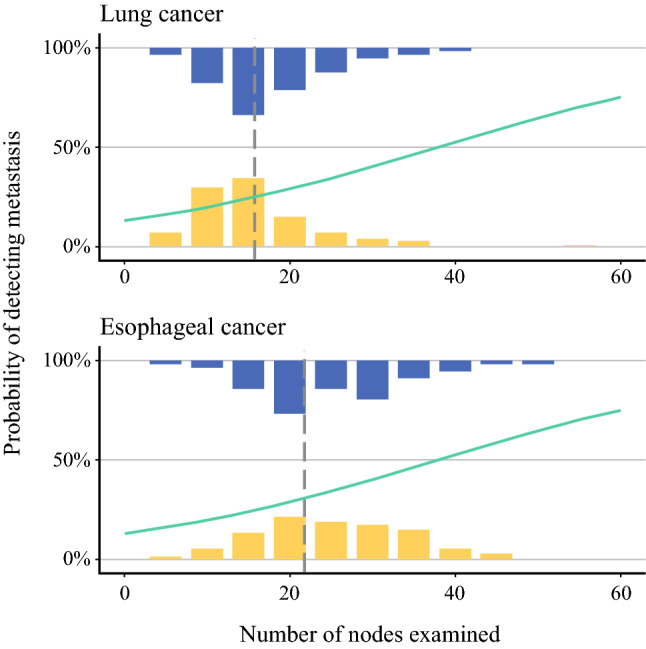
The results of predictive logistic modeling of 50% probability cut-off value of detecting metastasis adjusted by proportion of patients with metastasis detected. In lung cancer, the estimated cut-off value was 15.7 and in esophageal cancer 21.8

**Fig. 5:**
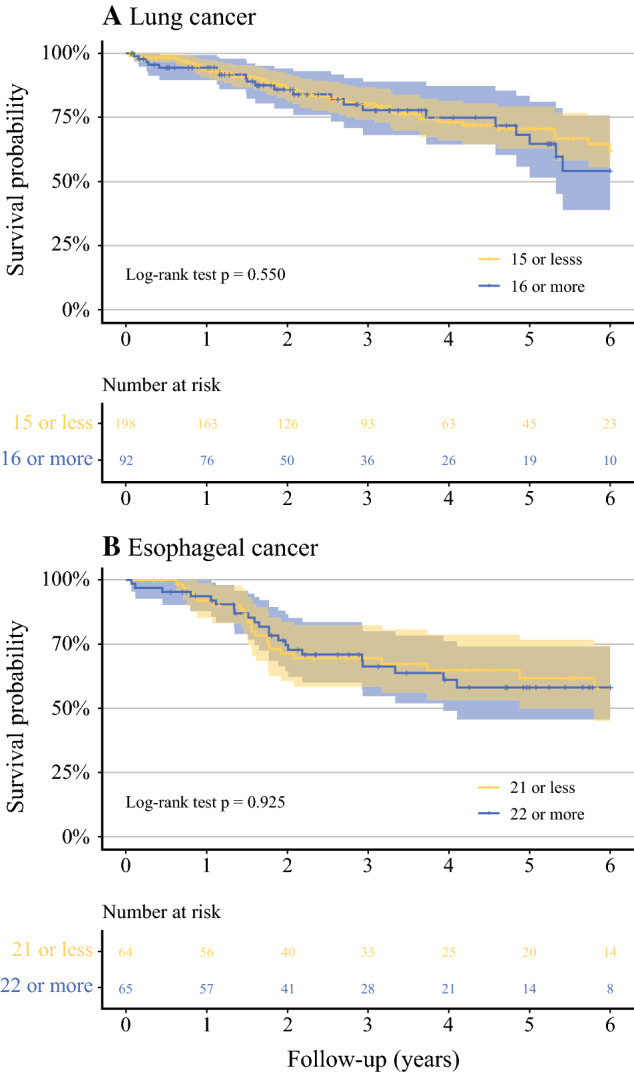
**A** and **B** Kaplan-Meier survival curves of lung and esophageal cancer patients stratified by 50% cut-off value of detecting metastasis

In lung cancer, the cut-off value for 50% probability of detecting metastasis calculated by predictive logistic modeling was 15.7 (Fig. 4). Patients with examined lymph nodes above and below this cut-off value had similar survival [log-rank *p* = 0.550; adjusted RMST ratio (more than cut-off/less than cut-off) 0.99, 95% CI 0.88–1.11, *p* = 0.833; Fig. 5a]. In the analysis of only pN0 lung cancers, patients with a lymph node count above and below the cut-off value had highly similar overall survivals (log-rank *p* = 0.984). Similar to overall survival, disease-specific survival in patient groups stratified by the thoroughness of the pathologists or the cut-off value for 50% probability of detecting metastases was comparable (Supplementary Fig. 3A).

### Esophageal Cancer

Of seven pathologists, four had a median reported lymph node value of 25 or more (Fig. 2). A significant difference existed in the number of examined lymph nodes between the pathologists (*p* < 0.001). The pathologist with the highest median number of examined nodes had 52.9% higher lymph node yield compared with the pathologist with the lowest median number of nodes in lung tissue (28 vs. 17, *p* = 0.003). No differences were detected in patient demographics or treatment between patients whose specimens were examined by pathologists having a high lymph node count compared with patients whose specimens were examined by pathologists having a low lymph node count (Supplementary Table 2).

Subgroups based on the thoroughness of pathologists had similar survivals (log-rank *p* = 0.987, Fig. 3B). Adjusted RMST ratio for < 25 vs. ≥ 25 lymph nodes was 0.95 (95% CI 0.79–1.15, *p* = 0.612).

In esophageal cancer, the 50% probability cut-off value of detecting metastasis was 21.8 (Fig. 4). Patients with examined lymph nodes above and below this cut-off value had no difference in overall survival (log-rank *p* = 0.925; adjusted RMST ratio 0.99, 95% CI 0.73–1.07, *p* = 0.216; Fig. 5B). Patient groups separated by the thoroughness of the pathologist or the cut-off for 50% probability of detecting metastases had comparable disease-specific survivals as well (Supplementary Fig. 3B).

## Discussion

This study revealed high interobserver variability between pathologists in the number of examined lymph nodes in both lung and esophageal cancer. In both cancers, the highest median number of examined lymph nodes was up to 50% higher than the lowest. By just narrowing this variability towards the median of all pathologists, a higher rate of patients would have been classified to have undergone optimal lymphadenectomy considered to enhance both staging and survival.^15^

The high detected variability in this study in the number of examined lymph nodes, as previously reported in colorectal cancer, seems to reflect individual preferences in pathologists’ working habits.^8–10^ This is supported by our study in both esophageal and lung cancer due to the detected variability regardless of the standardized lymphadenectomy and nodal station separation techniques by surgeons. Regardless of standardized lymphadenectomy techniques in bladder and colorectal cancers, high variation in lymph node counts has been observed between pathologists and institutions.^6–10^ Though optimization of specimen handling protocols has been shown to increase lymph node yield,^16^ setting a required minimum lymph node count may, however, tempt pathologists to also include tissue samples other than lymph nodes as ‘potential lymph nodes’ into the count.^17^ Overall, without a decrease in the high variability in the examined number of lymph nodes by pathologists, the role and reliability of lymph node yield can be questioned.^17^ Both internal and external quality improvement measures and education has been shown to improve pathologic lymph node examination in lung cancer.^18,19^ Overall, quality assurance recommendations for pathology include several important internal aspects as well as external validation.^20^

In the current study, the comparison of pathologists based on their thoroughness, determined by the examined lymph node count, did not show eligible evidence of difference in the mid-term survival of lung or esophageal cancer patients. In both of these cancers, though conflicting outcomes have been published,^21^ several studies have revealed an association between a higher number of examined lymph nodes and improved survival.^22–24^ The advantages of successful lymphadenectomy relate to more accurate cancer staging and more comprehensive excision of the disease in the form of metastatic lymph nodes.^24,25^ Any deficiency in lymph node examination by pathologists may bring into question the accuracy of staging and, especially in lung cancer, result in neglecting the need for an adjuvant therapy. Findings of the current study suggest, however, that the differences in survival reflect other factors than the examined lymph node count. Variation in the lymph node count, along with variation among pathologists, have been reported to be associated with tumor size, resected specimen size, surgical technique, and individual surgeon.^26^ Collaboration between a surgeon and a pathologist is important for optimal lymph node yield. From the perspective of the individual patient, in the light of this study, the surgeon has a key role that exceeds that of the pathologist in determining outcomes. In light of the weak association between lymph node count and survival in general and, in contrast, a stronger respective association in lymph node negative patients, lymph node count may not be a useful and reliable measure of lymphadenectomy quality, but rather a measure of the uncertainty of a pathology report.

Thus far, a consensus on the optimal lymph node yield in either lung or esophageal cancer has yet to be achieved. Highly varying numbers of examined lymph nodes with highly varying outcomes have been reported^27^. Previous studies and guidelines have recommended lymph node yield ranging from 10 to 16 nodes in lung cancer and 15 to 30 in esophageal cancer.^4,21,28–32^ Higher lymph node yield has been shown to increase the probability of detecting advanced disease.^25^ According to the 50% cut-off probability of detecting a metastasis by lymph node count in the current study, patients were divided into groups. In lung cancer, the cut-off was 15.7 and in esophageal cancer it was 21.8. The comparison between patients with the number of examined lymph nodes above and below these cut-offs revealed, however, that the differences in survival were rather modest. This finding was somewhat unexpected in the light of impaired survival of lung cancer patients with a missed metastasis in pathological examination or reported survival improvement in patients with a high lymph node count and a complete pathologic response after neoadjuvant therapy for esophageal cancer.^33,34^ This study by Lutfi et al. was, however, a multicenter study with a highly different distribution of lymph node count compared with our median count of 22.^34^ Their median count was well below 14.^34^ Furthermore, they first demonstrated a cut-off of seven as having a statistically significant survival improvement, while such a low count was rarely seen in our study. In this study, one surgeon performed all resections using a standardized quality, proven technique.^13,35^ Therefore, a negligible survival improvement with a high lymph node count seems to reflect extensive and uniform surgical lymphadenectomy. In the case of technical variability and lack of surgical standardization, as is often the case in retrospective multicenter studies, a lymph node count could have an effect on survival and can be considered as one of the quality measures.

Solutions to improve the quality of lymph node examination have been proposed in previous literature. It has been shown that standardization of lymph node sampling and specimen handling has a beneficial effect on pathological examination, leading to higher lymph node count, more appropriate lymph node station dissection, as well as more accurate grading of surgical healthy tissue margins.^36–39^ In addition, visual quality control, such as tissue sample photographing and video recording of lymph node dissection, have been suggested to improve lymph node examination quality.^40–42^ Therefore, implementation of a surgicopathological evaluation protocol is highly recommended.^13,43,44^

### Strengths and Limitations

There are several strengths in the current study. First, since only a single team was involved, surgical resection and lymphadenectomy was performed highly uniformly for all patients in accordance with international guidelines.^35^ Second, lymph nodes from different fields were separated into distinct specimens by surgeons already in the operating room, thus minimizing variability caused by specimen handling. Third, in the specimen examination, all pathologists evaluated both lung and esophageal cancer specimens. Fourth, since the study series included all esophageal resection patients, except salvage cases, and 88.3% of lung cancer resections at the population-level, the sample may be considered representative of lung and esophageal cancer patients suitable for surgical treatment. The main limitation of this study was its retrospective design, which predisposes the study results to selection bias and uncontrollable confounding. A limited number of patients meant that we could not perform extensive subgroup analyses by different tumor T- or N-stages.

## Conclusion

In our study, a high variability was detected in the number of examined lymph nodes between pathologists. However, no association with lymph node yield and mid-term survival of lung and esophageal cancer patients was observed. Outcomes were similar when comparing subgroups based on the examined lymph nodes or the cut-off number optimally detecting lymph node metastasis. These findings suggest that the quality of lymphadenectomy is not reliably graded solely based on the examined number of lymph nodes.

## Supplementary Information

Below is the link to the electronic supplementary material.

**A** and **B** Kaplan-Meier survival curves stratified by N-stage.. Supplementary file1 (TIFF 13605 kb)

**A** and **B** Kaplan-Meier curves showing cancer-specific survival stratified by N-stage. Supplementary file2 (TIFF 13605 kb)

**A** and **B** Kaplan-Meier curves showing cancer-specific survival of lung and esophageal cancer patients stratified by 50% cut-off value of detecting metastasis (adjusted RMST ratio [16 or more/15 or less] 0.94, 95% CI 0.86–1.01, *p* = 0.098 in lung cancer, and [22 or more/21 or less] 0.91, 95% CI 0.75–1.10; *p* = 0.310 in esophageal cancer). Supplementary file3 (TIFF 15820 kb)

Supplementary file4 (DOCX 28 kb)
