# Supplementary figures and images for: Standard Lymphadenectomy for Esophageal and Lung Cancer: Variability in the Number of Examined Lymph Nodes Among Pathologists and Its Survival Implication
Source: Ann Surg Oncol. 2022 Nov 25;30(3):1587–95. doi: 10.1245/s10434-022-12826-0 (PMC9908682; doi:10.1245/s10434-022-12826-0)

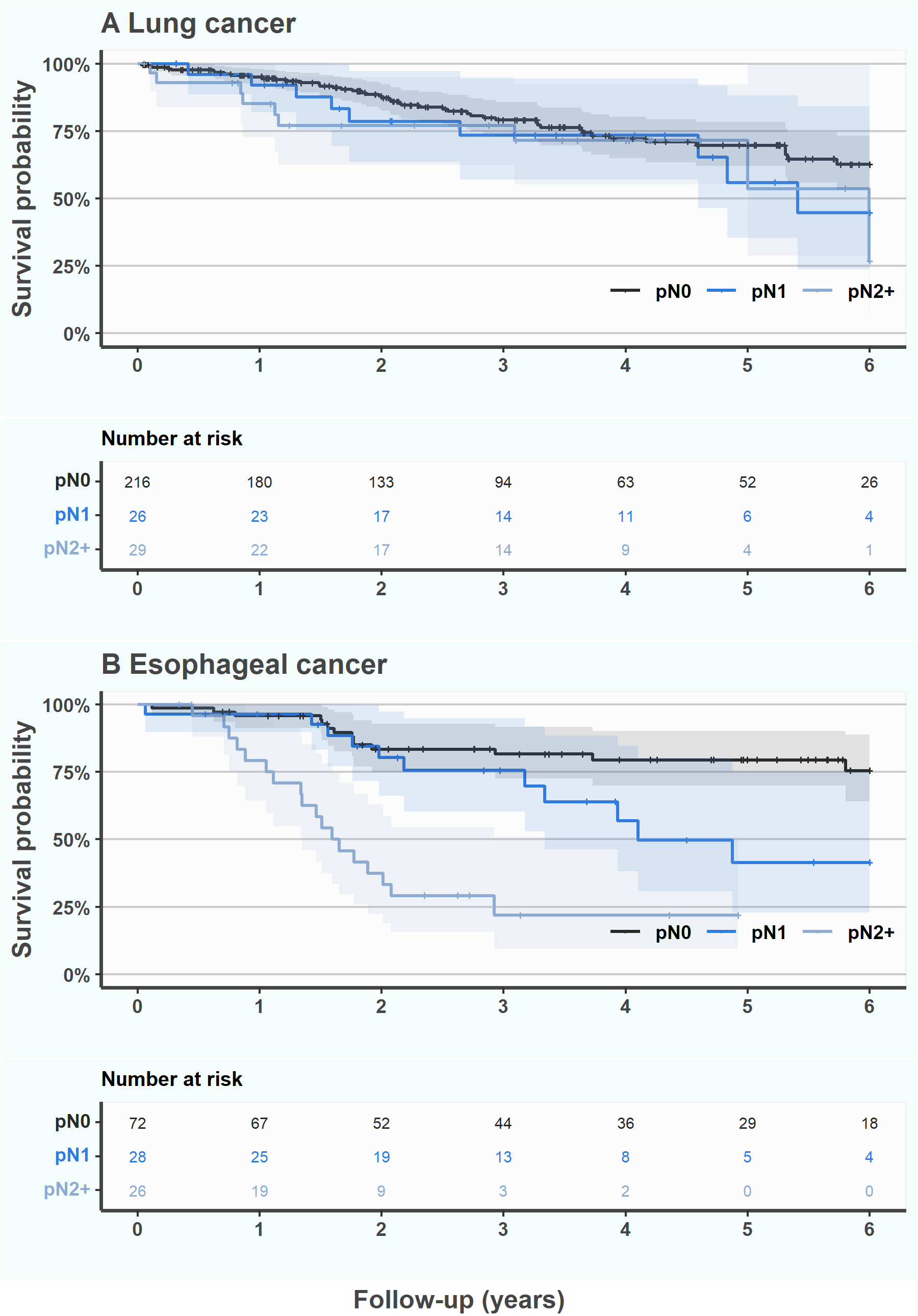

Supplement: Supplementary file 1 — A and B Kaplan-Meier survival curves stratified by N-stage.. Supplementary file1 (TIFF 13605 kb) [file 10434_2022_12826_MOESM1_ESM.tiff]

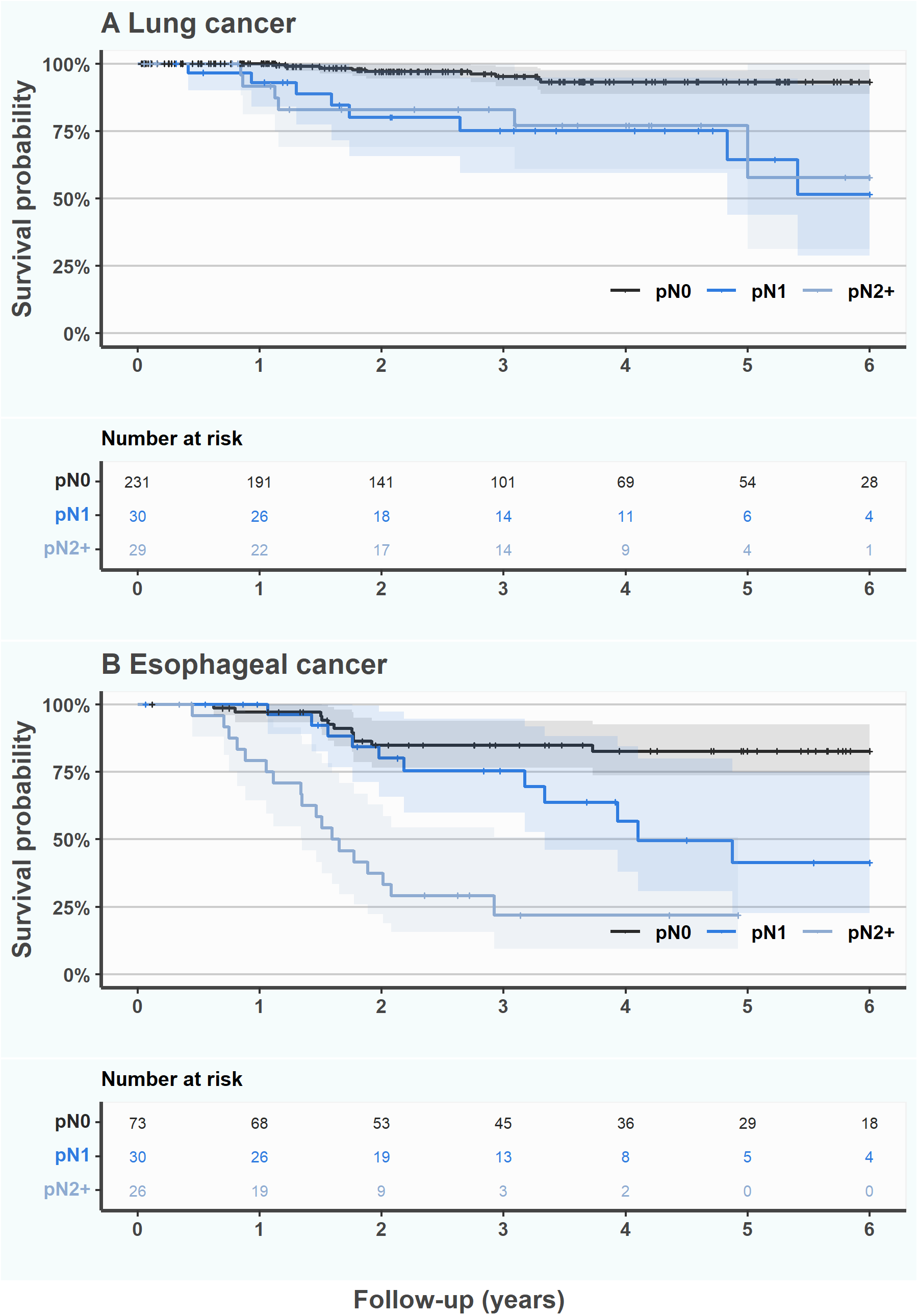

Supplement: Supplementary file 2 — A and B Kaplan-Meier curves showing cancer-specific survival stratified by N-stage. Supplementary file2 (TIFF 13605 kb) [file 10434_2022_12826_MOESM2_ESM.tiff]

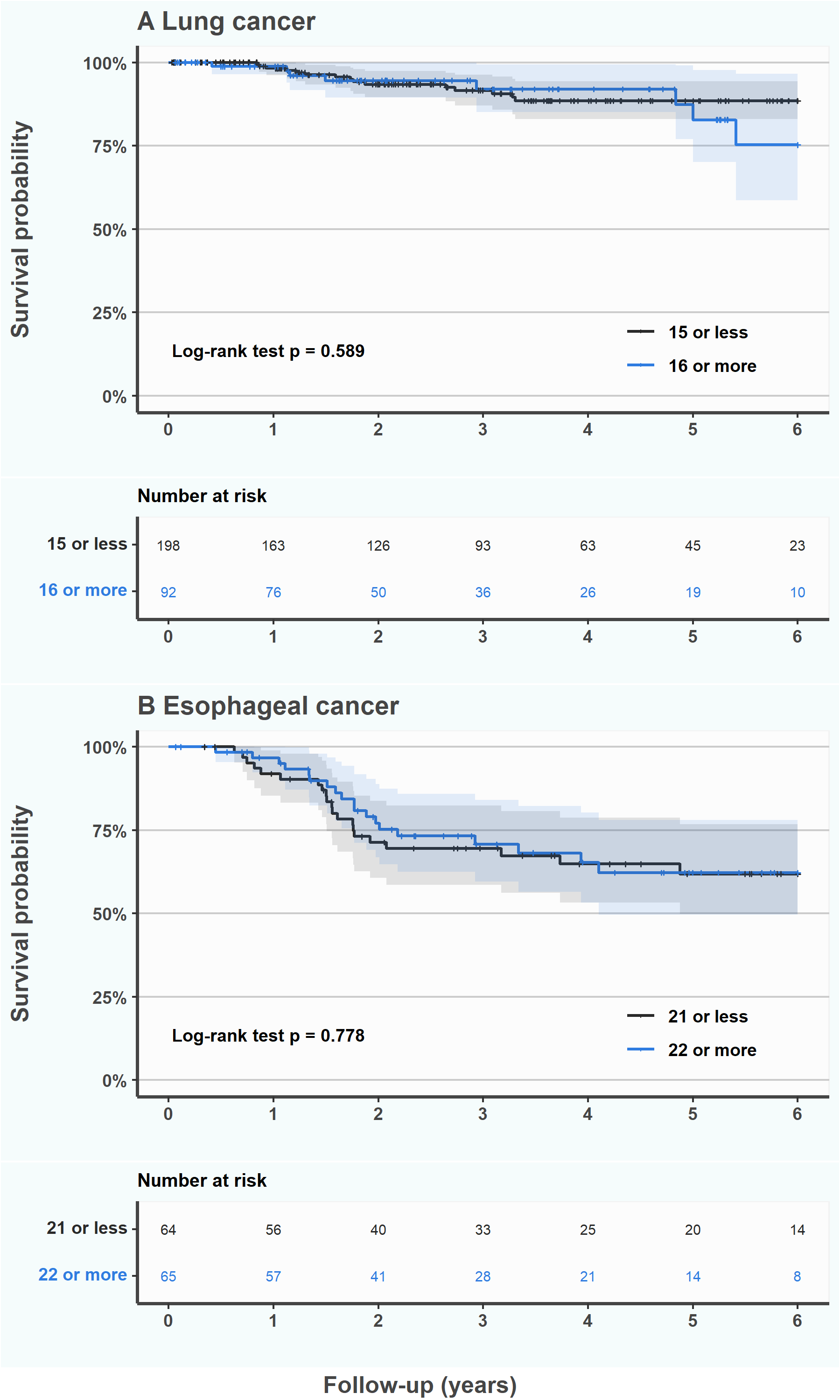

Supplement: Supplementary file 3 — A and B Kaplan-Meier curves showing cancer-specific survival of lung and esophageal cancer patients stratified by 50% cut-off value of detecting metastasis (adjusted RMST ratio [16 or more/15 or less] 0.94, 95% CI 0.86–1.01, p = 0.098 in lung cancer, and [22 or more/21 or less] 0.91, 95% CI 0.75–1.10; p = 0.310 in esophageal cancer). Supplementary file3 (TIFF 15820 kb) [file 10434_2022_12826_MOESM3_ESM.tiff]
