# Supplementary material for: Standard Lymphadenectomy for Esophageal and Lung Cancer: Variability in the Number of Examined Lymph Nodes Among Pathologists and Its Survival Implication
Source: Ann Surg Oncol. 2022 Nov 25;30(3):1587–95. doi: 10.1245/s10434-022-12826-0 (PMC9908682; doi:10.1245/s10434-022-12826-0)
Supplement: Supplementary file 4 — Supplementary file4 (DOCX 28 kb) [file 10434_2022_12826_MOESM4_ESM.docx]

**Supplementary tables**

**Supplementary table 1.** Characteristics of lung cancer patients with tissue samples examined by pathologists with high and low median lymph node count.

|  | High LNC | Low LNC | p |
| --- | --- | --- | --- |
|  | (n = 116) | (n = 159) |  |
| Sex male, n (%) | 72 (62.1) | 101 (63.5) | 0.905 |
| Age, mean (SD) | 70.07 (7.46) | 69.21 (9.61) | 0.425 |
| BMI, mean (SD) | 26.21 (4.18) | 25.98 (4.77) | 0.679 |
| Side left, n (%) | 50 (43.1) | 68 (42.8) | 1.000 |
| Resection extent, n (%) |  |  | 0.905 |
| Segmentectomy | 45 (38.8) | 64 (40.3) |  |
| Lobectomy | 71 (61.2) | 95 (59.7) |  |
| VATS | 19 (83.6) | 101 (87.4) | 0.473 |
| Charlson Comorbidity Index, n (%) |  |  | 0.112 |
| 0-1 | 55 (47.4) | 90 (56.6) |  |
| 2-4 | 53 (45.7) | 65 (40.9) |  |
| >4 | 8 (6.9) | 4 (2.5) |  |
| Performance status (stair climbing), n (%) |  |  | 0.827 |
| <10m | 6 (5.2) | 11 (6.9) |  |
| 10-14m | 15 (12.9) | 16 (10.1) |  |
| >14m | 95 (81.9) | 132 (83.0) |  |
| cStage, n (%) |  |  | 0.241 |
| 1 | 74 (63.8) | 115 (72.3) |  |
| 2 | 29 (25.0) | 27 (17.0) |  |
| 3-4 | 13 (11.2) | 17 (10.7) |  |
| LNC = lymph node count |  |  |  |

**Supplementary table 2.** Characteristics of esophageal cancer patients with tissue samples examined by pathologists with high and low median lymph node count.

|  | High LNC | Low LNC | p |
| --- | --- | --- | --- |
|  | (n = 68) | (n = 59) |  |
| Sex male, n (%) | 47 (69.1) | 48 (81.4) | 0.168 |
| Age, mean (SD) | 66.10 (11.92) | 66.24 (10.27) | 0.948 |
| BMI, mean (SD) | 25.39 (4.49) | 26.30 (5.53) | 0.316 |
| Charlson Comorbidity Index, n (%) |  |  | 0.577 |
| 0-1 | 50 (73.5) | 48 (81.4) |  |
| 2-4 | 18 (26.5) | 11 (18.6) |  |
| Performance status (stair climbing), n (%) |  |  | 0.993 |
| <14m | 6 (8.8) | 5 (8.5) |  |
| ≥14m | 62 (91.3) | 54 (91.5) |  |
| cStage, n (%) |  |  | 0.417 |
| 1 | 18 (26.5) | 10 (17.0) |  |
| 2 | 49 (72.1) | 49 (83.1) |  |
| 3-4 | 1 (1.5) | 0 (0.0) |  |
| Neoadjuvant treatment, n (%) |  |  | 0.620 |
| Preoperative chemotherapy | 15 (22.1) | 16 (27.1) |  |
| Preoperative chemoradiotherapy | 33 (48.5) | 32 (54.2) |  |
| LNC = lymph node count |  |  |  |
